# Time dependency of foamy virus evolutionary rate estimates

**DOI:** 10.1186/s12862-015-0408-z

**Published:** 2015-06-26

**Authors:** Pakorn Aiewsakun, Aris Katzourakis

**Affiliations:** Department of Zoology, University of Oxford, Oxford, OX1 3PS UK

**Keywords:** Foamy virus, Time dependent rate phenomenon, Evolutionary rate dynamics, Power law

## Abstract

**Background:**

It appears that substitution rate estimates co-vary very strongly with their timescale of measurement; the shorter the timescale, the higher the estimated value. Foamy viruses have a long history of co-speciation with their hosts, and one of the lowest estimated rates of evolution among viruses. However, when their rate of evolution is estimated over short timescales, it is more reminiscent of the rapid rates seen in other RNA viruses. This discrepancy between their short-term and long-term rates could be explained by the time-dependency of substitution rate estimates. Several empirical models have been proposed and used to correct for the time-dependent rate phenomenon (TDRP), such as a vertically-translated exponential rate decay model and a power-law rate decay model. Nevertheless, at present, it is still unclear which model best describes the rate dynamics. Here, we use foamy viruses as a case study to empirically describe the phenomenon and to determine how to correct rate estimates for its effects. Four empirical models were investigated: (i) a vertically-translated exponential rate decay model, (ii) a simple exponential rate decay model, (iii) a vertically-translated power-law rate decay model, and (iv) a simple power-law rate decay model.

**Results:**

Our results suggest that the TDRP is likely responsible for the large discrepancy observed in foamy virus short-term and long-term rate estimates, and the simple power-law rate decay model is the best model for inferring evolutionary timescales. Furthermore, we demonstrated that, within the Bayesian phylogenetic framework, currently available molecular clocks can severely bias evolutionary date estimates, indicating that they are inadequate for correcting for the TDRP. Our analyses also suggest that different viral lineages may have different TDRP dynamics, and this may bias date estimates if it is unaccounted for.

**Conclusions:**

As evolutionary rate estimates are dependent on their measurement timescales, their values must be used and interpreted under the context of the timescale of rate estimation. Extrapolating rate estimates across large timescales for evolutionary inferences can severely bias the outcomes. Given that the TDRP is widespread in nature but has been noted only recently the estimated timescales of many viruses may need to be reconsidered and re-estimated. Our models could be used as a guideline to further improve current phylogenetic inference tools.

**Electronic supplementary material:**

The online version of this article (doi:10.1186/s12862-015-0408-z) contains supplementary material, which is available to authorized users.

## Background

It has been noted that evolutionary rates calculated over short timescales, such as those calculated from population genetic data, are much greater than those calculated across geological time frames, such as species evolutionary rates. This discrepancy between short-term and long-term rates is very widespread in nature, noticed in both viral genes [1–6] and cellular genes, including bacterial genes [7, 8], mitochondrial genes of worms [9], insects [10, 11], fish [12, 13], birds [14–16], and primates [17–23], as well as nuclear genes of various multicellular organisms [24–27] (see [28] for a review, and references therein). Further investigation has revealed that, in fact, the value of the rate estimate does not vary discretely, but continuously decreases as the measurement timescale increases [28]. This ‘time dependent rate phenomenon’ (TDRP) was first demonstrated in cellular genes [20]. Subsequently, by pooling substitution rate estimates of diverse viruses together, Duchêne et al. [29] showed that the rate estimates of RNA and DNA viruses also exhibit this pattern.

To date, the processes that lead to the TDRP are still very much unclear. Many hypotheses have been proposed to explain it, such as temporal changes in organismal biology and natural selection pressure [28]. Numerous methodological artefacts also have the potential to systematically bias the rate estimates in such a way that short-term rates will appear to be much greater than the long-term ones [28]. Since the rate of evolution is a central component of evolutionary study, an accurate evolutionary inference requires that the TDRP is integrated into the analysis. An ideal approach to this problem is to understand how the TDRP is generated, and improve evolutionary inference tools so that they can account for the factors underlying the TDRP. However, given a large number of potential underlying factors and our current incomplete understanding of their interactions, untangling and explicitly accounting for each of them individually would be impractical at present [20, 30].

One pragmatic approach to this problem is to infer evolutionary timescales by using an empirical model describing the relationship between rate estimates and their measurement timescales. This approach has been employed in several studies (e.g. [12, 20]). A number of empirical models, such as the vertically-translated exponential rate decay model and the power-law rate decay model, have been proposed, and used, for TDRP correction in evolutionary inferences [12, 20, 31]. Nevertheless, at present, it is still unclear which model best describes the phenomenon, and to answer this question we require substitution rate estimates that are computed over various timeframes. These can be obtained from a dataset of molecular sequences for which several divergence dates are known; the more divergence dates available, the more suitable the dataset for this purpose. Furthermore, it is also preferable that the dates are distributed relatively evenly across the entire evolutionary timescale that is being examined [32]. In this work, we seek to empirically describe the TDRP in detail and explore the various patterns of rate decay over time by using foamy viruses (FVs) as a case study.

FVs are a group of complex retroviruses that have a very stable and long co-speciation history with their hosts, stretching back more than a hundred million years [33–35], and because of this, almost all of their divergence dates can be directly inferred from those of their hosts [36–38]. Based on this co-speciation and the known divergence dates of their primate hosts, the long-term rate of evolution of FVs has been estimated to be ~7.79 × 10^-9^ to 1.7 × 10^-8^ nucleotide substitutions per site per year (s/n/y) [33, 39]. This is much slower than rates of substitution of other RNA viruses, typically reported to be in range of 10^-3^ to 10^-4^ s/n/y [6, 40, 41]. The high similarity observed between extant FVs and their ancient endogenous counterparts [34, 35, 42, 43] has also lent further support to this notion of slow-evolving FVs. Altogether, FVs are thereby widely regarded as one of the most slow-evolving RNA viruses currently known [44].

These slow long-term rates of FV evolution stand in sharp contrast to their high mutation rate. *In vitro* analyses have shown that the FV replication error rate (5.8 × 10^-5^ s/n/replication) is comparable to that of human immunodeficiency virus (HIV) (6.5 × 10^−5^ s/n/replication) [45], which is one of the fastest-evolving viruses ever documented. This fast FV mutation rate has also been confirmed in human embryonic cell lines, where the *in vivo* rate was calculated to be at least 1.1 × 10^-5^ s/n/replication [46]. Moreover, by following a population of African Green Monkey FVs for 9 years, it was estimated that the FV evolutionary rate is as high as ~3.75 × 10^-4^ s/n/y [47]. This short-term rate is ~4-5 orders of magnitude higher than the long-term rate counterparts, estimated under the FV-host co-speciation assumption.

The fact that almost all of the divergence dates of FVs can be directly inferred from those of their hosts makes FVs an ideal system to study the TDRP. Here, we use 14 extant FVs (Additional file 1: Table S1) as a case study to present direct evidence of a smooth decay of nucleotide substitution rate estimates as the measurement timescale increases. We also empirically describe the rate decay pattern, examine whether or not the TDRP can explain the discrepancy between FV short-term and long-term rates, and discuss the applications and limitations of our empirical rate decay models, as well as how the TDRP may bias evolutionary inference and rate estimate interpretation.

## Results

### FV nucleotide substitution rate estimate decreases with measurement timescale

To compute FV nucleotide substitution rates for various timescales, we first estimated FV phylogenies using Pol protein (1,116 aa) and *pol* nucleotide (3,351 nt) alignments under the Bayesian and maximum-likelihood frameworks. The aligned sequences were checked for recombination, but no significant evidence was found, both at the nucleotide (*p* = 0.266) and protein (*p* = 0.357) levels. (See Methods for details.) Our results show that all phylogenies are perfectly in agreement with one another topologically (Additional file 2: Figure S1), and also consistent with the results from previous studies [33–35]. We thus considered the estimated tree topology as our best working hypothesis, and used it to estimate FV nucleotide substitution rates.

**Fig. 1:**
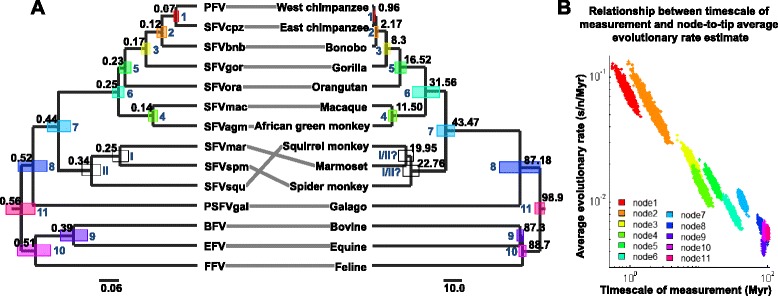
Foamy virus (FV) and corresponding host phylogenies, and the relationship between node-to-tip average evolutionary rate estimate and timescale of measurement. (**a**, left) FV phylogeny (taxon definitions and GenBank sequence accession numbers are in Additional file 1: Table S1). Black numbers are estimated total per-lineage nucleotide substitutions in the units of substitutions per site. The node bars represent the uncertainties of the estimated nucleotide substitution divergences. The scale bar is in units of substitutions per site. (**a**, right) Host tree. Black numbers are estimated divergence dates in units of millions of years, for which the estimation uncertainties are shown by node bars. The scale bar is in units of millions of years. The topology of the host tree and the divergence dates were estimated elsewhere (see the references in Additional file 1: Table S2). Solid lines between the two trees indicate FV-host associations, and blue Arabic numbers (**1**-**11**) indicate matching FV-host nodes (i.e. FV-host co-speciation events). Nodes within the FV tree that could not be mapped conclusively onto the host tree are labelled by blue Roman numbers (**I** and **II**). The colours of the node bars on the FV tree correspond to the colours of the bars on the host tree. **b** Timescales of rate measurement and node-to-tip average evolutionary rate estimates on a log-log scale. The node numbers (**1**-**11**) refer to those in the FV and host trees (**a**). The colours correspond to the node bars’ colours. The summary of the raw data can be found in Additional file 1: Table S2

We first estimated node-to-tip total per-lineage nucleotide substitutions (*s* estimates) from the *pol* nucleotide alignment under the fixed FV phylogeny, and the Bayesian phylogenetic framework. We employed a strict molecular clock with a fixed rate of 1 to obtain branch lengths in units of substitutions per site. A strict clock was applied (i.e. the tips were forced to align) under the assumption of consistency among rate estimates calculated using different nodes and tips. Thus, our study can also be viewed as an attempt to correct for the TDRP given the strict clock assumption. In total, 13 posterior distributions of *s* estimates were obtained, one for each internal node, and we could assign timescales (*t* estimates) to 11 of them based on the FV-host co-speciation history. This, in turn, allowed us to compute 11 distributions of node-to-tip average substitution rates ($\overline{r}$ estimates). (See Methods for details.) A summary of the results can be found in Fig. 1, and Additional file 1: Table S2. Preliminary linear regression analyses suggest that log-transformed $\overline{r}$ is significantly negatively correlated with log-transformed *t* (linear regression: correlation coefficient [95 % highest probability density (HPD)] = -0.577 [-0.614, -0.542]; randomisation test: number of randomization tests = 15,000, number of data points for null distribution construction in each test = 100, p < 0.01 in all 15,000 tests; See Methods for details).

### Empirical description of FV nucleotide substitution rate decay

It has been proposed that a vertically-translated exponential decay function (Eq. 1) is a good empirical description for the apparent decay of the instantaneous substitution rate (*r*) [20]. This model has been employed in several studies for TDRP correction (e.g. [31]). However, an examination of mitochondrial DNA control regions of cichlids showed that a simple power law function (Eq. 4) can also empirically describe the phenomenon reasonably well [12]. To systematically explore these hypotheses, we examine another two empirical functions by varying whether the exponential and power law functions are vertically translated: a simple exponential rate decay function (Eq.2) and a vertically-translated power-law function (Eq. 3). The four models can be expressed mathematically as follows:

1 $$ {r}_{VEX}={\alpha}_{VEX}{e}^{-t{\beta}_{VEX}}+{k}_{VEX} $$

2 $$ {r}_{EX}={\alpha}_{EX}{e}^{-t{\beta}_{EX}} $$

3 $$ {r}_{VPL}={\alpha}_{VPL}{t}^{-{\beta}_{VPL}}+{k}_{VPL} $$

4 $$ {r}_{PL}={\alpha}_{PL}{t}^{-{\beta}_{PL}} $$

where *t* (unit time, ut) is the measurement/evolutionary timescale in the present-to-past direction, where *t* = 0 is the present; *k* (s/n/ut) is the stable long-term rate of evolution parameter; and *α* and *β* are arbitrary model parameters. “*VEX*”, ‘*EX*’, ‘*VPL*’, and ‘*PL*’ subscripts indicate the model to which the parameters and variables belong: vertically-translated exponential rate decay (VEX), simple exponential rate decay (EX), vertically-translated power-law rate decay (VPL) and simple power-law rate decay (PL) models, respectively. Note that, since a strict clock was applied and the tips were all aligned, the timescales of the rate measurement can be interpreted as node heights/divergence dates and vice versa.

To investigate how well each model describes the FV $\overline{r}$ dynamics, we first derived four equations depicting the relationship between *s* and *t* for the VEX (Eq. 5), EX (Eq. 6), VPL (Eq. 7), and PL (Eq. 8) models based on Eq. 1–4, respectively, as follows;

5 $$ {s}_{VEX}={\displaystyle {\int}_{t=0}^{t=t}}{r}_{VEX}dt=\frac{\alpha_{VEX}}{\beta_{VEX}}\left(1-{e}^{-t{\beta}_{VEX}}\right)+t{k}_{VEX} $$

6 $$ {s}_{EX}={\displaystyle {\int}_{t=0}^{t=t}}{r}_{EX}dt=\frac{\alpha_{EX}}{\beta_{EX}}\left(1-{e}^{-t{\beta}_{EX}}\right) $$

7 $$ {s}_{VPL}={\displaystyle {\int}_{t=0}^{t=t}}{r}_{VPL}dt=\frac{\alpha_{VPL}{t}^{1-{\beta}_{VPL}}}{1-{\beta}_{VPL}}+t{k}_{VPL} $$

8 $$ {s}_{PL}={\displaystyle {\int}_{t=0}^{t=t}}{r}_{PL}dt=\frac{\alpha_{PL}{t}^{1-{\beta}_{PL}}}{1-{\beta}_{PL}} $$

The curves were forced to go through the origin to conform the expectation that there are no substitutions at time equal to zero. We next simply divided both sides of the equations by *t* to derive equations describing the relationship between $\overline{r}$ and *t* for the four respective models (Eq. 9–12, respectively):

9 $$ \frac{s_{VEX}}{t}={\overline{r}}_{VEX}=\frac{\alpha_{VEX}}{t{\beta}_{VEX}}\left(1-{e}^{-t{\beta}_{VEX}}\right)+{k}_{VEX} $$

10 $$ \frac{s_{EX}}{t}={\overline{r}}_{EX}=\frac{\alpha_{EX}}{t{\beta}_{EX}}\left(1-{e}^{-t{\beta}_{EX}}\right) $$

11 $$ \frac{s_{VPL}}{t}={\overline{r}}_{VPL}=\frac{\alpha_{VPL}{t}^{-{\beta}_{VPL}}}{1-{\beta}_{VPL}}+{k}_{VPL} $$

12 $$ \frac{s_{PL}}{t}={\overline{r}}_{PL}=\frac{\alpha_{PL}{t}^{-{\beta}_{PL}}}{1-{\beta}_{PL}} $$

**Fig. 2:**
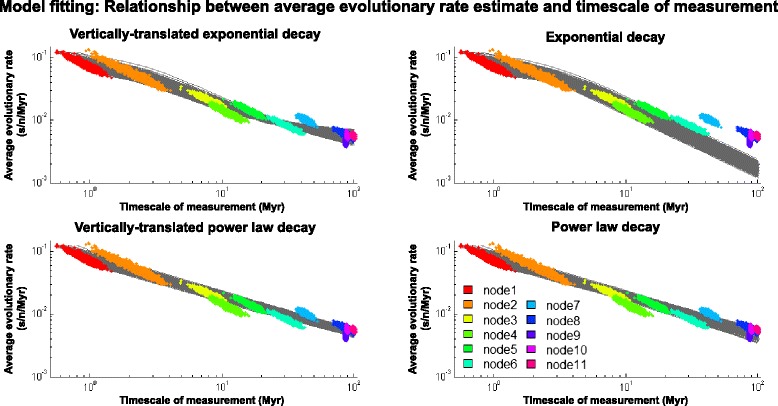
Empirical description of the relationship between rate measurement timescale (*t*) and average evolutionary rate estimate ($\overline{r}$). Eq. 9 (top-left), Eq. 10 (top-right), Eq. 11 (bottom-left), and Eq. 12 (bottom-right) were fitted to 1,500 randomly-sampled sets of corresponding $\overline{r}$ and *t* estimates under the criterion of the least sum of squared errors of $\overline{r}$ (grey lines). The colours correspond to those of the node bars in Fig. 1a. The node numbers refer to those on the trees in Fig. 1a. The summary of the results can be found in Additional file 1: Table S3

We then fitted all four models to the $\overline{r}$ and *t* estimates, and assessed how well the models describe the data by using adjusted R^2^ (${\overline{R}}^2$) scores. Although it is clear from visual inspection that the EX model tends to underestimate long-term rates (Fig. 2), overall, all four models seem to describe the data well, indicated by their high ${\overline{R}}^2$ scores (${\overline{R}}^2$ score [95 % HPD]: Eq. 9: 0.99 [0.95, 1.00]; Eq. 10: 0.97 [0.94, 0.99]; Eq. 11: 0.98 [0.92, 1.00]; Eq. 12: 0.98 [0.93, 1.00]). The results are shown in Fig. 2, and Additional file 1: Table S3.

In addition, we also recovered FV short-term and long-term rate estimates (calculated over a timescale of 10 years and 30 million years (Myr), respectively) by using these four models. The VEX and EX models estimated the short-term rate of FVs to be ~10^-7^ to 10^-8^ s/n/y (median rate estimates [95 % HPD]: VEX: 1.06 × 10^-7^ [7.43 × 10^-8^, 1.51 × 10^-7^]; EX: 9.68 × 10^-8^ [6.92 × 10^-8^, 1.35 × 10^-7^]). This is much lower than the previously reported FV short-term rate estimate (~3.75 × 10^-4^ s/n/y [47]). On the other hand, the VPL and PL models estimated the rate to be in the order of 10^-4^ to 10^-5^ s/n/y (median rate estimates [95 % HPD]: VPL: 8.17 × 10^-5^ [3.24 × 10^-5^, 2.20 × 10^-4^]; PL: 7.53 × 10^-5^ [3.24 × 10^-5^, 1.45 × 10^-4^]), comparable to the previously reported FV short-term rate estimate. In contrast, all four models calculated the long-term rate to be ~10^-8^ to 10^-9^ s/n/y (median rate estimates [95 % HPD]: VEX: 8.75 × 10^-9^ [8.16 × 10^-9^, 9.39 × 10^-9^]; EX: 5.99 × 10^-9^ [4.58 × 10^-9^, 7.00 × 10^-9^]; VPL: 9.97 × 10^-9^ [9.02 × 10^-9^, 1.10 × 10^-8^]; PL: 9.93 × 10^-9^ [8.58 × 10^-9^, 1.09 × 10^-8^]), all comparable to the established long-term rates of FVs (~7.79 × 10^-9^ [39] to 1.7 × 10^-8^ [33] s/n/y).

### Leave-one-out cross validation analyses

**Fig. 3:**
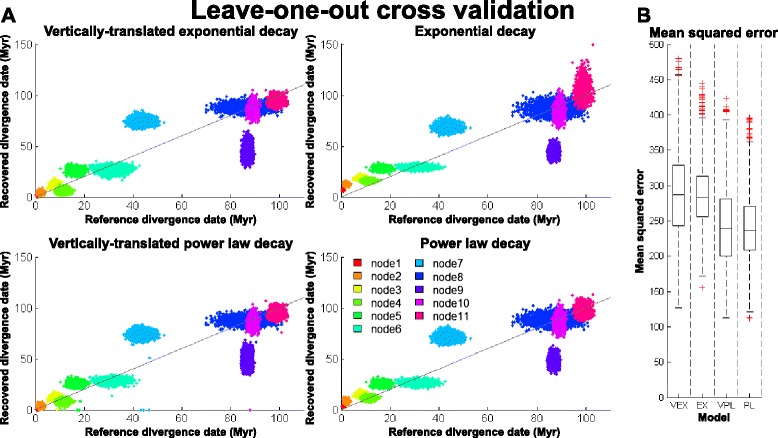
Leave-one-out cross validation (LOOCV) tests. The models were validated under the context of divergence date (*t*) inference, by using LOOCV tests (see Methods for details). In (**a**), the recovered *t* values are shown against the reference *t* values, inferred under the co-speciation assumption. The colours of the data points correspond to the colours of the node bars in Fig. 1a. The node numbers refer to those in the trees in Fig. 1a. The grey lines show where the recovered *t* values are exactly equal to the references. The out-of-sample mean squared errors are shown in (**b**). A summary of the results can be found in Additional file 1: Table S4. VEX: vertically-translated exponential rate decay model; EX: simple exponential rate decay model; VPL: vertically-translated power-law rate decay model; and PL: simple power-law rate decay models

Although the high ${\overline{R}}^2$ scores suggest that all four models can describe the relationship between $\overline{r}$ and *t* estimates well (Additional file 1: Table S3), it has been noted that ${\overline{R}}^2$ scores are inappropriate for comparing the performance of nonlinear models, and can severely bias model selection in favour of models with more parameters [48]. Corrected Akaike information criterion (AICc) and Bayesian information criterion (BIC) have been suggested to be more suitable for this purpose [48]. However, the calculation of AICc and BIC scores requires likelihood functions of the models. Since our models are empirical, derived based on a top-down approach, we lack such information. Given these limitations and constrains, we thus used the leave-one-out cross validation (LOOCV) technique to compare our models, in the context of their ability to recover *t* values given the *s* values. Eq. 5–8 were used in these analyses as they depict how *t* relates to *s*. The *t* values inferred under the FV-host co-speciation assumption were used as references, and by comparing the recovered *t* values against them, we computed out-of-sample mean squared error (MSE_OOS_) scores. These scores were used as a measurement of the overall predictability of the models. (See Methods for details.) The results are summarised in Fig. 3 and Additional file 1: Table S4.

Overall, we found that the *t* values recovered by all four models are largely comparable to the references (Fig. 3a), indicative of high predictability for all four models. This finding also implies that the co-speciation hypothesis is itself internally consistent. Two discrepancies were found between the reference and recovered *t* values however. For all four models, we found that (i) the recovered time to most recent common ancestor (tMRCA) of simian FVs (SFVs) (~68.87-74.50 Myr, node **7**, Fig. 1a) are much greater than the corresponding host tMRCA (~43.47 Myr, [38]), and (ii) that the recovered tMRCA of bovine FV (BFV) and equine FV (EFV) (~43.06-48.85 Myr, node 9, Fig. 1a) are much lower than the respective host tMRCA (~87.3 Myr, [36]). To verify that these estimates are not correlated (i.e. that one did not cause the other), we re-calculated the tMRCAs for these two FV groups using all four models estimated independently of nodes **7** and **9**. Both discrepancies could still be observed (data not shown), indicating that they are not artefacts.

Interestingly, unlike other models, the EX model in particular seems to have a tendency to overestimate shallow divergence dates (Fig. 3a; top right). This is at odds with the observation that the EX model has a tendency to incorrectly describe the long-term rates (Fig. 2; top right). Systematic differences in the distribution of the data points describing the $\overline{r}$-*t* and *t*-*s* relationships may explain this discrepancy. While the variance of $\overline{r}$ estimates is greatest when *t* is low, the variance of *t* estimates is greatest when *s* is high. Thus, the influence of the data points in parameter estimation differed when the EX model was fitted to the two datasets; that is, while Eq. 10 parameter estimation (depicting the $\overline{r}$-*t* relationship) was primarily influenced by the data points near the *y* axis, Eq. 6 parameter estimation (depicting the *t*-*s* relationship) was primarily influenced by the data points further away from the *y* axis. This problem is not as apparent in the other three models however, suggesting that they suffered to a much lesser extent from this effect.

By comparing the MSE_OOS_ scores, we found that the PL model (Eq. 8) has the least MSE_OOS_ overall (MSE_OOS_ [95 % HPD] = 236.25 [146.21, 325.40] Myr, mean rank = 1.62; Fig. 3b), indicating that it is the best model for inferring *t*, and thus most preferable as a TDRP-correcting tool. Interestingly, the VPL model (Eq. 7) was found to be the second best model (MSE_OOS_ [95 % HPD] = 239.04 [148.06, 357.06] Myr, mean rank = 1.87; Fig. 3b), suggesting that the extra parameter *k*_*VPL*_ in the VPL model does not significantly improve, but instead over-parameterises the model. Likewise, the VEX model (Eq. 5) was found to be the worst model (MSE_OOS_ [95 % HPD] = 287.52 [169.43, 400.58] Myr, mean rank = 3.32; Fig. 3b), and the EX model (Eq. 6) was found to be the second worst model (MSE_OOS_ [95 % HPD] = 282.79 [204.40, 369.84] Myr, mean rank = 3.18; Fig. 3b).

### Examining the performance of currently available molecular clocks in TDRP correction

Several relaxed-clock models have been developed to address the problem of rate variation among lineages [49–51]. These models allow rates to vary among branches, and thus over time; therefore, they have the potential to be used as a tool for correcting for the TDRP in evolutionary inferences. In this section, we explored how well currently available relaxed-clock models can accommodate the TDRP under the context of FV timescale inference. We chose to explore scenarios where only three nodes are available as calibrating nodes as they represent realistic circumstances where calibrating information is limited. Nodes **7** and **9** were excluded from this analysis. This is because the LOOCV analyses show that the reference and the recovered *t* values differ greatly, and it is not possible to determine which is closer to the true values.

Two schemes of date calibration were examined: (i) *aggregated-node calibration* scheme, where all three calibrating nodes are of similar timescales, and (ii) *dispersed-node calibration* scheme where calibrating nodes are of different timescales. In the former calibration scheme, we explored three different scenarios: (i) shallow- (*t* range: ~0.96-8.30 Myr; nodes **1**, **2**, and **3**), (ii) intermediate- (*t* range: ~11.50-31.56 Myr; nodes **4**, **5**, and **6**), and (iii) deep-timescale calibration scheme (*t* range: ~87.18-98.90 Myr; nodes **8**, **10**, and **11**). Similarly, we explored three (arbitrary) circumstances for the latter calibration scheme: (i) dispersed-I calibration scheme (*t* range: ~2.17-87.18 Myr; nodes **2**, **5**, and **8**), (ii) dispersed-II calibration scheme (*t* range: ~0.96-88.7 Myr; nodes **1**, **4** and **10**), and (iii) dispersed-III calibration scheme (*t* range: ~8.30-98.9 Myr; nodes **3**, **6**, and **11**). Three currently available molecular clocks were investigated, including (i) a strict molecular clock, (ii) a log-normal relaxed clock [51], and (iii) a random-local relaxed clock [50]. The strict clock was included to examine how the TDRP would affect *t* inferences if it is ignored. We focused on Bayesian timescale estimates, and used the *pol* nucleotide alignment that was used in the phylogenetic reconstruction to estimate the timescales (see Methods). Again, the *t* estimates inferred under the FV-host co-speciation assumption were used as reference *t* estimates.

**Fig. 4:**
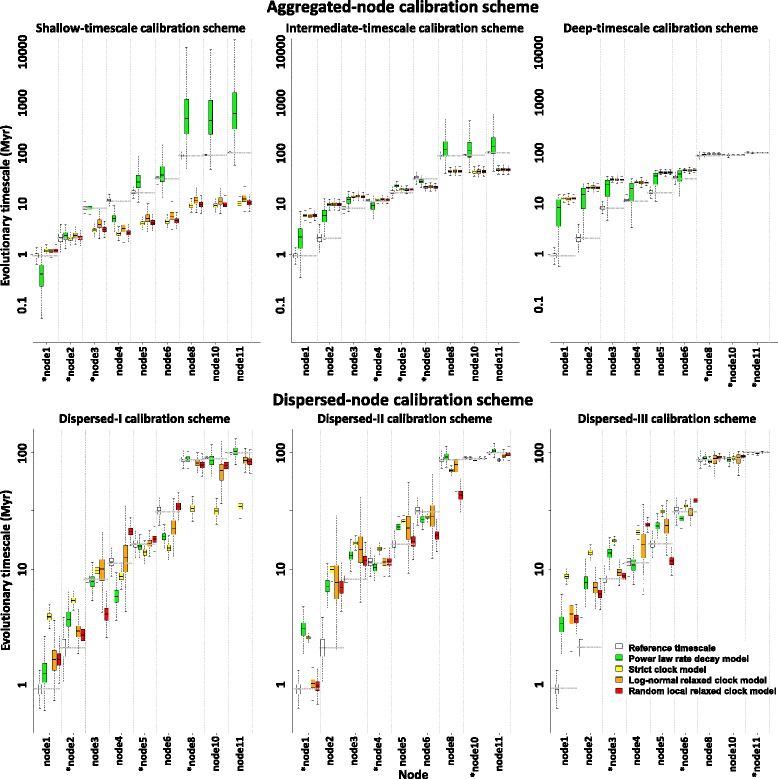
Performance of various molecular clocks in accommodating the time-dependent rate phenomenon (TDRP). We explored the performance of various clock models in accommodating the TDRP in the context of foamy virus (FV) evolutionary timescale inference under various hypothetical scenarios, where only three nodes are available as calibrating nodes. Two calibration schemes were examined: (i) aggregated-node calibration scheme (top), in which all three calibrating nodes are of similar timescales, and (ii) dispersed-node calibration scheme (bottom), in which calibrating nodes are of different timescales. The aggregated-node calibration scheme was sub-divided into three sub-schemes: (i) shallow-timescale calibration scheme (top left), (ii) intermediate-timescale calibration scheme (top middle), and (iii) deep-timescale calibration scheme (top right). The dispersed-node calibration scheme was also sub-divided into three arbitrary sub-schemes: (i) dispersed-I calibration scheme (bottom left), (ii) dispersed-II calibration scheme (bottom middle), and (iii) dispersed-III calibration scheme (bottom right). Four sets of dates were plotted from different clock implementations and one of our TDRP models: (i) simple power rate decay model (green), (ii) strict clock model (yellow), (iii) log-normal relaxed clock model (orange), and (iv) random-local relaxed clock model (red). FV evolutionary timescales inferred from the host timescales (reference timescales) are in white. Horizontal dotted grey lines are median reference timescales. The results are plotted on a log scale, and arranged in such a way that the evolutionary timescales of shallow nodes are on the left, and those of deep nodes are on the right. The node numbers refer to those in Fig. 1a. Calibrating nodes are indicated by asterisks ‘*’. The summary of the results can be found in Additional file 1: Table S5 & S6

We also compared these currently available clocks to our PL model (Eq. 8). Unlike the above however, the calculation was not done under a full Bayesian phylogenetic framework. Rather, we used *t* and *s* estimates of the calibrating nodes to estimate the model, and then inferred the *t* values of other nodes based on their *s* estimates. The results are shown in Fig. 4, and Additional file 1: Table S5.

#### Aggregated-node calibration scheme

Under the aggregated-node calibration scheme (Fig. 4; top), our results show that the *t* values estimated under the strict clock, log-normal relaxed clock, and random-local relaxed clock are comparable to one another. This is likely because the calibrating nodes do not provide enough signal for the rate variation among lineages to be estimated. Indeed, we found that the coefficients of rate variation estimated under the two relaxed clock models do not deviate very far from zero (Additional file 1: Table S6).

Overall, we found that the *t* values of the nodes adjacent to the calibrating nodes are estimated relatively accurately, comparable to the reference values. However, as we move further away from the calibrating nodes, the *t* estimates become increasingly more inaccurate. Under the shallow-timescale calibration scheme, the *t* values of deep nodes are severely underestimated (Fig. 4; top left). In contrast, the *t* estimates of shallow nodes are severely overestimated under the deep-timescale calibration scheme (Fig. 4; top right). In the intermediate-timescale calibration scheme, the *t* values of deep nodes are underestimated while those of shallow nodes are overestimated (Fig. 4; top middle). Our results are consistent with the findings in a previous study that solely focused on SFVs [52].

In contrast, our results showed that the PL model could accommodate the TDRP reasonably well under these examined scenarios, better than the currently available molecular clocks. Although we found that the uncertainties of *t* estimates are extremely large (thus providing relatively little information about the actual dates themselves), the reference *t* values are almost always contained within the *t* distributions estimated under the PL model, whereas those estimated under the current clock models do not even overlap with the reference *t* distribution (Fig. 4; top). The median *t* estimates from the PL model are also closer to the reference values, which is most apparent in the intermediate- and deep-timescale calibration schemes (Fig. 4; top middle and right, respectively), but less so in the shallow-timescale calibration scheme (Fig. 4; top left). These large uncertainties are unlikely an intrinsic property of the PL model however, but a shortcoming of our extrapolation approach in which the *t* and *s* values are not estimated in conjunction with one another. Coupled with the fact that these are extrapolations over large timescales and that the uncertainty of the *s* estimate increases down the tree (Fig. 1a), it is unsurprising to find that this problem is most apparent in the shallow-timescale calibration scheme (Fig. 4; top left), but less so in the intermediate-timescale calibration scheme (Fig. 4; top middle), and least in the deep-timescale calibration scheme (Fig. 4; top right). These findings thus indicate that deep nodes are preferable as calibrating nodes, consistent with the results from a previous simulation study that investigated the impact of calibrating node position on timescale estimates [52].

#### Dispersed-node calibration scheme

Under the dispersed-node calibration scheme (Fig. 4; bottom), we found that the performances of the log-normal and random-local relaxed clocks differ from that of the strict clock. This is likely because the calibrating nodes are spread out in time and hence provide adequate information for the rate variation among branches, and thus over time, to be modelled more accurately. Indeed, the coefficients of rate variation estimated under the two relaxed clocks deviate far from zero (Additional file 1: Table S6).

Overall, we found that the strict clock model performs worst, and the *t* values obtained under the clock vary substantially between sub-schemes. For example, in the dispersed-I calibration scheme (Fig. 4; bottom left), the *t* values of intermediate nodes were estimated relatively accurately, but those of shallow nodes were overestimated, and those of deep nodes were underestimated. On the other hand, in the dispersed-II and -III calibration schemes (Fig. 4; bottom middle and left, respectively), the *t* values of deep nodes were estimated relatively accurately whereas those of shallow nodes were severely overestimated. In contrast, the two relaxed clock models seem to perform reasonably well, returning *t* values that are comparable to the references, and also to those inferred under the PL model. This, in turn, indicates that the performance of these three models is comparable under these scenarios. Moreover, we also observed that the *t* distributions inferred under the PL model are tighter compared to those inferred under the aggregated-node calibration scheme. This is likely because the dispersed-node schemes involve interpolation rather than extrapolation.

### Inferring missing FV divergence dates by using the PL model

Consistent with the results from previous studies [35, 53], amidst the stable FV-host co-speciation history, we found a few mismatches in the history of New World monkey (NWM) FVs and their hosts (Fig. 1a). These involve (i) the split between marmoset and spider monkey FVs (SFVmar and SFVspm, respectively) (Fig. 1a; node I), and (ii) the divergence leading to squirrel monkey FV (SFVsqu) lineage (Fig. 1a; node II).

As a case study, we used the PL model to infer the *t* values for these two nodes. To do so, Eq. 8 was fitted to the dataset of *t* and *s* estimates, with those of nodes **7** and **9** excluded (as we could not conclusively determine the true *t* values for these two nodes; see above). The models were then used to infer the *t* values of node **I** and **II** from their *s* estimates. Our analyses inferred the tMRCA of SFVmar and SFVspm to be ~23.40 [19.25-27.52] Myr, and the earlier branching of SFVsqu lineage to occur ~40.81 [35.58-46.08] Myr ago.

## Discussion

### What are the causes of the TDRP?

Our analyses show that the estimated values of the FV substitution rates decrease continuously with measurement timescales (Fig. 1), and the PL model is the best model for correcting for the TDRP. It is possible that the TDRP observed here is the result of changes in FV biology, such as polymerase fidelity, replication speed, and/or transmission modes. These ideas have been put forward as plausible explanations for the rate variations observed in several viruses, including hepadnaviruses [3], and human T-cell lymphotopic viruses [4, 5] as well as RNA viruses in general [6]. These hypotheses would be reasonable and sensible if the values of the rate estimates changed discretely. However, as the rate estimate continuously decreases with the measurement timescale, this would posit that the biology of FVs has been continuously changing with time in such a way that that the viral rate of evolution gradually increases through time. Moreover, since the TDRP is not unique to FVs, but very common among viruses, these hypotheses would also imply that the biology of many viruses has changed in a similar manner. Although possible, this is extremely unlikely [54]. We therefore believe that changes in viral biology likely do not play a major role in governing the overall decay trend of viral evolutionary rate estimates.

Changes in natural selection pressure have also been put forward as a potential underlying cause of the TDRP [27, 29, 55]. Nevertheless, like viral biology, it is extremely difficult to imagine that environmental factors would have changed systematically with time so that viruses experience less and less purifying selection pressure as they evolve. It is important to note that we are by no means suggesting that changes in environment do not cause viral evolutionary rates to alter. Indeed, they can, and this has been observed. An analysis of bat rabies viruses has shown that their evolutionary rates are strongly correlated with the host local environments, suggesting that environmental changes can alter the rate of viral evolution [56]. However, we argue that, for the alteration of environment and/or natural selection to play a major role in generating the TDRP, not only must they change the rate, but the effect must also be time-correlated, which we consider unlikely. We thus believe that, rather than reflecting a genuine change in natural selection pressure, the observed TDRP is likely an artefact caused by other factors.

So, if viral biology, environmental factors, and selection pressure remain relatively constant through time, what could possibly cause the TDRP? It has been proposed that short-term rate estimates tend to be overestimated due to the inclusion of transient deleterious variations [28, 41, 57], recent adaptive changes [58–60], and sequencing errors [20, 61–63]. Errors in coalescent calibration information have also been suggested as a sufficient explanation for elevated short-term rate estimates [64]. In contrast, misspecification of substitution model [65, 66], saturation of nucleotide changes [14, 28, 65, 67], and improper accounting for rate heterogeneity among sequence positions [30] can lead to underestimation of long-term rate estimates. All of these factors could contribute to the TDRP, and at present, the importance and relevance of each factor is still poorly understood and continues to be debated (see [28] for a review).

### The TDRP as a possible explanation of the short-term and long-term rate discrepancy

A large discrepancy of ~4-5 orders of magnitude has been observed between FV short-term and long-term rate estimates. Indeed, such discrepancies have been found not only in FVs, but many and diverse RNA and DNA viruses [3–5, 54, 60]. Rather than viewing them as conflicting rate estimates, it is has been proposed that this discrepancy may result from estimating the rates over different extremes of the TDRP [54].

In order to investigate whether the TDRP can explain this discrepancy or not, we used our four rate models to recover the short-term and long-term rate estimates of FVs. Our analyses showed that all of the four models could recover the long-term rate relatively well. However, given that the models were parameterised on FV long-term rate estimates, this is expected and unsurprising. In contrast, we found that only the VPL and PL models could recover the FV short-term rate accurately, but the VEX and EX models severely underestimated the rate by ~3-4 orders of magnitude. These results further support the use of the PL model as a tool for TDRP correction, and simultaneously indicate that, indeed, the short-term and long-term rate discrepancy in FVs can be explained by the TDRP.

### Implications of the TDRP

There are a number of implications of the TDRP. One of them is that it is important to take the timescale of rate measurement into consideration when using or interpreting evolutionary rate estimates; otherwise, the outcomes could be severely biased. For example, it is inappropriate to use long-term rate estimates to infer or evaluate viral short-term epidemiological dynamics, as they could give an erroneous impression that the viruses being considered are of low adaptive and cross-species transmission potential. Similarly, in the specific case of FVs, which are candidate gene-carrying vectors for gene-therapy [68, 69], using their long-term rates to evaluate the risks of FV-derived gene vectors could be misleading, as they are in fact capable of evolving at a (short-term) rate as high as ~3.75 × 10^-4^ s/n/y [47], comparable to those of highly pathogenic and fast-adapting viruses like HIVs [41, 70–72] and influenzas [41, 73–75].

Another important, and perhaps more obvious, implication is that it will no longer be valid to naïvely extrapolate rate estimates across different time frames when inferring evolutionary timescales. The assumption of a single molecular clock can bias the timescale inference with the severity increasing with the timescale of rate extrapolation. As the value of the rate estimate continuously decreases with the measurement timescale, the TDRP should appear in, and is relevant to, every phylogenetic analysis. That is, if two or more evolutionary rate estimates are calculated over different timescales from a particular phylogeny (e.g. use different internal nodes to calibrate the rate), the TDRP should show up. Nevertheless, in practice, if the timescale of evolutionary investigation is short, e.g. hundreds of years (which are typical for infectious disease analyses), the uncertainty of the rate estimate may overwhelm the effect of the TDRP, and the phenomenon might not be observed. Indeed, the strict molecular clock has sometimes been identified as appropriate for studies over short timescales such as epidemiological studies [41, 76, 77], but not for longer timescale analyses such as evolutionary investigations that compare viruses in different host species [4, 78, 79]. Moreover, the TDRP can also bias demographic parameter estimations such as effective population sizes and migration rates if it is unaccounted for. This is simply because the calculation involves estimating or knowing substitution rates [28, 80].

Similar to the results from previous studies [52, 81], our results show that evolutionary timescales calibrated under currently available molecular clocks are highly sensitive to the choice of calibrating nodes. The effect is most pronounced when the calibrating nodes are of the same timescale (Fig. 4; top). As short-term rate estimates are greater than the long-term ones, calibrating deep divergence dates with shallow nodes will underestimate them. Conversely, using deep nodes to calibrate shallow divergence dates will tend to overestimate them. Strikingly, we found that the current relaxed clocks do not perform any better than the strict clock under these circumstances, indicating that they are not an effective solution to the TDRP problem. Compared to the currently available relaxed-clock models, the PL model performs better and is more consistent across the calibration schemes, even when calibrating nodes are of similar timescales (Fig. 4; top).

Our results suggest that the currently available relaxed-clock models suffer much less from the TDRP problem, and perhaps are equally good to the PL model, if calibrating nodes are dispersed in time (Fig. 4; bottom), consistent with the results from a previous study [32]. Nevertheless, in most realistic applications, the number and dispersal of calibrating nodes is serendipitous, dictated by their limited availability which often involves only one or a few nodes. Given this limited availability of time-calibrating information and the sensitivity of the current clocks to the choice of calibrating nodes, our results overall suggest that the current relaxed-clock models might not be an effective and practical solution to the TDRP problem yet. We, thus, believe that our PL model will be useful as a guideline to further improve our current evolutionary inference tools.

### Possible evolutionary rate dynamics heterogeneity among viral lineages

Our analyses showed that the tMRCA estimates of SFVs recovered by the four models are considerably higher than the host tMRCA (Fig. 3a and Additional file 1: Table S4; node **7**). Our analyses also estimated the tMRCA of SFVmar and SFVspm (~23.40 Myr) and that of NWM FVs (~40.81 Myr) to be greater than those of their hosts (~22.76 Myr, [38]). One possibility is that the tMRCAs inferred by our models are not artefacts, but resemble the real dates. This would however imply duplications of viral lineages in the absence of host diversification, which we consider to be unlikely. An alternative explanation is that the evolutionary rates of NWM FVs and the stem lineage are higher than average, and our analysis framework did not take this into account. Since we employed a strict clock to estimate *s* values, we thereby assumed that all taxa evolve under the same time-dependent rate dynamics. Thus, the incongruences between the predicted and inferred NWM FV divergence dates may be indicative of the heterogeneity of evolutionary rate dynamics among viral lineages, and this may bias evolutionary timescale inferences if it is unaccounted for. A solution to this problem would be to fit multiple time-dependent rate models to different parts of the tree.

Conversely, our results show that the recovered BFV/EFV tMRCAs are considerably lower than the host tMRCA (Fig. 3a and Additional file 1: Table S4; node 9), implying a cross-species FV transmission between equine and bovine hosts ~53-59 Ma. Unlike the scenarios discussed above, this is relatively reasonable and should not be ruled out. Alternatively, it could be that the substitution rate on the BFV-EFV stem branch is greater than that of other FVs. Further resolution of these questions would require the identification and analysis of FV genomes of other bovines and equines.

## Conclusions

Our knowledge of viral natural history has been greatly advanced by molecular analyses. One of the key steps in viral evolutionary study involves estimating the rate of substitution. By using FVs as a case study, we show that their evolutionary rate estimates are negatively correlated with the timescale of rate measurement, and this is likely responsible for the short-term/long-term rate discrepancy observed in FVs, and perhaps other viruses as well [54]. We also demonstrate that currently available relaxed-clock models are inadequate for accommodating the TDRP; using them to infer evolutionary timescales can severely bias the date estimates especially when rate-calibrating nodes are of similar timescales. We believe that the PL model developed here will be useful as a guideline for the further improvement of existing analytical tools. Our results also suggest that heterogeneity in rate dynamics among viral lineages may exist, and can affect evolutionary inference.

Combined, our work highlights the importance of the TDRP and heterogeneity in evolutionary rate dynamics among viral lineages. Given the potential impacts of the TDRP on evolutionary inference and rate estimate interpretation, the fact that it is so widespread in nature but has been noticed only recently could mean that the credibility of evolutionary timescale estimates of many viruses may need to be reconsidered.

## Methods

### Phylogenetic reconstruction

Four phylogenies of 14 extant foamy viruses (FVs) (Additional file 1: Table S1) were estimated from manually-curated Pol protein (1,116 aa; Additional file 3) and *pol* nucleotide (3,351 nt; Additional file 4) alignments. Potential recombination among aligned sequences was assessed with a quartet-based recombination detection program VisRD3 [82], both at nucleotide and protein levels. In both cases, the null distribution was built based on 1,000 datasets of randomly-shuffled sequences, and the extended statistical geometry (Hamming) weighting option was applied. In the nucleotide analysis, the window and step size were 200 and 40 nt, respectively, and in the protein analysis, the window and step size were 100 and 20 aa, respectively. The results showed no significant evidence for recombination. The best-fit amino-acid and nucleotide substitution models used in the phylogenetic reconstructions were determined to be rtREV + I + Γ(4) + F and GTR + I + Γ(4) by ProtTest 2.4 [83] and Jmodeltest 2.1.1 [84], respectively, under the AICc criterion.

The phylogenies were constructed under both the Bayesian and maximum-likelihood phylogenetic frameworks, by using MrBayes 3.2.1 [85] and MEGA 5.2 [86], respectively. In the Bayesian analyses, 2 independent MCMCs were run for 50,000,000 steps each, with the initial 12,500,000 steps discarded as burn-in. Parameters were thereafter logged every 2,500 steps. Metropolis coupling was applied, using the setting of 3 hot and 1 cold chains. Parameter estimate convergences were diagnosed using potential scale reduction factors (PSRFs). PSRFs of all parameters are ~1.000, indicating that they were all well sampled from their posterior distributions and had converged. In the maximum-likelihood analyses, bootstrap support values were calculated using 1,000 pseudoreplicates. A molecular clock was not imposed in either of the analyses. In total, four phylogenies were estimated: (i) a maximum-likelihood Pol protein tree, (ii) a Bayesian Pol protein maximum clade credibility (MCC) tree, (iii) a maximum-likelihood *pol* nucleotide tree, and (iv) a Bayesian *pol* nucleotide MCC tree (Additional file 2: Figure S1), all of which show the same topology.

Note that we did not include endogenous mammalian FVs – SloEFV [34], PSFVaye [35, 42], and ChrEFV [35, 43] – in the analysis. The reason is that their evolutionary rate is a mixed rate, comprising the rate of viral evolution and the neutral rate of host evolution, which could bias the analysis. Even if we can decompose the rate into the two rate components, it is still unclear how the ‘truncated’ rates of viral evolution would fit into the dynamics of the evolutionary rate of extant viruses. As a result, we focus our study on the rate dynamics of extant FVs only.

### Inferring FV node-to-tip total per-lineage substitutions (*s* estimates), evolutionary timescales (*t* estimates), and node-to-tip average evolutionary rates ($\overline{r}$ estimates) over various time frames, and assessing the correlation between $\overline{r}$ and *t* estimates

We estimated *s* values from the manually-curated *pol* nucleotide alignment under the Bayesian framework, by using BEAST 1.7.4 [87]. The strict molecular clock assumption with a fixed rate of 1, and Yule speciation process were applied. The topology of the phylogeny was fixed according to the phylogeny obtained in the phylogenetic reconstruction. The MCMC was run for 50,000,000 steps, with the initial 12,500,000 steps discarded as burn-in. Parameters were logged every 2,500 steps. In total, 15,000 sets of parameter estimates were sampled. Parameter value convergence and sampling independency were manually inspected using Tracer v1.5 [88]. We found all parameters had an effective sample size (ESS) of >350, indicating that all of them were well sampled and had converged.

In total, as there were 13 internal nodes, 13 posterior distributions of *s* estimates were obtained, 11 of which could be assigned to independently estimated evolutionary timescales of their hosts on the basis of the FV-host co-speciation assumption (Fig. 1a, and Additional file 1: Table S2). We then divided *s* estimates by their evolutionary timescale (*t* estimates) to derive $\overline{r}$ estimates for various time frames. Note that, since a strict clock was applied and the tips were all aligned, the timescale of the rate estimation is equivalent to the node heights in units of time. To accommodate the uncertainty of *t* and $\overline{r}$ estimates, we simulated 15,000 sets of *t* values under the assumption that they are normally distributed, and randomly paired them to each of the sub-datasets of *s* estimates to compute 15,000 sets of $\overline{r}$ estimates. The means of the *t* distributions were assumed to be equal to the median *t* estimates reported in the literature, and their standard deviations were calculated from the reported upper- and lower-bounds of the corresponding 95 % highest posterior density intervals: max $\left(\frac{Median- Lower\kern0.75em 95\%\kern0.75em HPD\kern0.75em  limit}{1.96},\kern0.5em \frac{Upper\kern0.75em 95\%\kern0.75em HPD\kern0.75em  limit- Median}{1.96}\right)$. The *t* simulation was constrained by the estimated phylogeny in such a way that the *t* values of child nodes were always lower than those of the corresponding parental nodes. This approach to model parameter estimation takes into account the uncertainty of *s*, *t*, and $\overline{r}$ estimates to the full extent, considering the whole space of their estimated Bayesian posterior distributions.

To preliminarily evaluate the correlation between $\overline{r}$ and *t*, we fitted a linear model to each of the 15,000 sub-datasets of the log-transformed $\overline{r}$ and *t* estimates, using the *LinearModel.fit* function implemented in MATLAB R2012a [89]. However, it has been noted that a correlation analysis between a quotient and its denominator has a tendency to yield a seemingly significant but in fact spurious negative correlation [90]. Randomisation tests have been recommended as a way to address this issue [90]. Here, we randomly matched *s* and *t* estimates to compute $\overline{r}$ values under the null hypothesis that there is no correlation between *s* and *t*, and in turn computed a ‘null’ $\overline{r}$-*t* correlation coefficient. In each of the 15,000 sub correlation analyses, this process was repeated 100 times to construct a distribution of the null $\overline{r}$-*t* correlation coefficient, which was then used to compute the p-value.

We also note that our data is not phylogenetically independent; for example, each of the terminal branches are included in several *s*, *t*, and $\overline{r}$ estimates. The results thus should be interpreted with an understanding that the data does not fully conform to the ordinary model-fitting assumption. It is also important to note that this problem is not unique to just the above analysis, but also applies to all subsequent model estimations in our study. In our model framework, this is inevitable, unfortunately, due to the fact that a phylogenetic independent dataset, i.e. a dataset of instantaneous rate estimates and their corresponding timescale, is extremely difficult, if not impossible, to obtain for FVs. However, validating the models under the context of *t* inference still shows that they work well despite this issue (see LOOCV analyses), indicating that our analyses suffer from this problem only to a low degree.

### Describing the temporal dynamics of $\overline{r}$

Four equations depicting the *t*-$\overline{r}$ relationship were derived based on four empirical rate decay hypotheses: (i) vertically-translated exponential rate decay hypothesis (Eq. 9), (ii) simple exponential rate decay hypothesis (Eq. 10), (iii) vertically-translated power-law rate decay hypothesis (Eq. 11), and (iv) simple power-law rate decay hypothesis (Eq. 12). These equations were in turn fitted to 1,500 datasets of corresponding *t* and $\overline{r}$ estimates randomly sampled from their posterior distribution under the criterion of the least sum of squared errors (LSE) of $\overline{r}$, using the *lsqcurvefit* function implemented in MATLAB R2012a [89]. All parameters were constrained to be greater than zero. ${\overline{R}}^2$ scores were used to preliminarily assess how well the models describe the data.

The estimated models were also used to recover previously reported short-term rate [47] and long-term rate [33, 39] estimates of FVs. In this study, the short-term and long-term rate estimates were defined and calculated over a timescale of 10 years, and 30 Myr, respectively.

### Validating the performance of the models under the context of *t* inference by using the leave-one-out cross validation (LOOCV) technique

We first derived four equations describing the relationship of *t* and *s* from the four empirical rate reduction hypotheses (Eq. 5–8, see Results), and fitted them to 1,500 sets of corresponding *t* and *s* estimates, sampled from their posterior distributions consisting of 15,000 datasets. For consistency, we constructed the *t*-*s* dataset in such a way that it corresponded to the dataset of *t* and $\overline{r}$ estimates used in the $\overline{r}$ dynamics analyses. In total, the models were validated for 1,500 rounds. In each round, the dataset was partitioned into a testing set containing an estimate of *s* and *t* of one particular node, and a training set containing the rest of the data. The models were fitted to the training set under the criterion of the LSE of *t*, by using the *lsqcurvefit* function implemented in MATLAB R2012a [89]. All parameters in the models were constrained to be greater than zero. The resultant models were then used to infer the *t* of the testing node from its *s* estimate. This process was repeated such that the *t* and *s* of every node is used exactly once as the testing data to complete one round of LOOCV testing. The overall performance of the models was assessed based on the out-of-sample mean squared error (MSE_OOS_), and the *t* values inferred under the FV-host co-speciation assumption were used as references. To compare the models, we applied Friedman’s test to the MSE_OOS_, and performed a post-hoc analysis (complete-pairwise MSE_OOS_ comparisons based on Wilcoxon signed-rank tests) with the Bonferroni multiple-testing correction. The significance was evaluated at α = 0.05.

### Examining the effect of the TDRP on *t* inference

We examined the effect of the TDRP on *t* inference under six hypothetical scenarios, in all of which only three nodes were used as calibrating nodes (see Results). Nodes **7** and **9** (Fig. 1a) were excluded from the analysis. The *t* values inferred under the FV-host co-speciation assumption were used as references. Three currently available molecular clocks were examined: (i) a strict molecular clock, (ii) a log-normal relaxed clock [51], and (iii) a random-local relaxed clock [50]. The *t* inference was performed under the Bayesian phylogenetic framework, implemented in BEAST 1.7.4 [87], by using the *pol* nucleotide alignment that was used in the phylogenetic reconstruction. The Yule speciation process and GTR + I + Γ(4) substitution model were applied. This model was determined to be the best for our alignment by Jmodeltest 2.1.1 [84] under the AICc criterion. The topology of the phylogeny obtained in the phylogenetic reconstruction was fixed. The MCMC was run for 50,000,000 steps, with the initial 12,500,000 steps discarded as burn-in. Parameters were logged every 2,500 steps. Parameter value convergence and sampling independency were manually inspected using Tracer v1.5 [88]. For the runs that returned parameters with ESSs of <200 (a recommended cut-off [51]), we repeated the analyses and combined the results until the ESSs of all parameters were >200.

Moreover, we also examined the PL model for how well it addresses the TDRP under these various hypothetical scenarios. The *t* and *s* estimates of the calibrating nodes were used to compute the model parameters. For consistency, the dataset of *t* and *s* estimates used in this investigation was the same one that was used in the LOOCV analyses. The model was fitted to each of the 1,500 sub-datasets using the *lsqcurvefit* function implemented in MATLAB R2012a [89] under the criterion of the LSE of *t*, and the parameter estimation was constrained so that all parameters were greater than zero. The estimated model was then used to infer the *t* values of other nodes based on their *s* estimates.

### Inferring missing FV divergence dates by using the PL model

The PL model was fitted to the dataset of *t* and *s* estimates that was used in the LOOCV analyses under the criterion of the LSE of *t*, but without the data associated with nodes **7** and **9**. The fitting was performed using the *lsqcurvefit* function implemented in MATLAB R2012a [89], and was constrained so that all parameter values were greater than zero. The estimated model was then used to infer the missing *t* values for two nodes based on their *s* estimates: (i) node **I** (the split between marmoset and spider monkey FVs) and (ii) node **II** (the basal diversification of all New World monkey FVs).

### Availability of supporting data

The data sets supporting the results of this article are included within the article (and its additional files).

## Additional files

Additional file 1: Table S1. GenBank accession numbers of foamy virus (FV) nucleotide sequences. **Table S2.** Foamy virus (FV) divergence dates (*t* estimates), node-to-tip total per-lineage nucleotide substitutions (*s* estimates), and average evolutionary rates ($\overline{r}$ estimates). **Table S3.** Model fitting – relationship between average evolutionary rate estimate and measurement timescale. **Table S4.** Summary of the results from leave-one-out cross validation analyses. **Table S5.** Summary of the results from the examination of the effect of the time-dependent rate phenomenon on evolutionary timescale inference. **Table S6.** Coefficients of rate variation. Additional file 2: Figure S1. Foamy virus (FV) phylogenies. Four phylogenies of 14 extant FVs were constructed based on Pol protein (**left column**) and *pol* nucleotide (**right column**) alignments under the Bayesian (**top row**) and maximum-likelihood (**bottom row**) framework (See Methods for details). Numbers on nodes are node supports (**top row**: Bayesian posterior support; **bottom row**: bootstrap support), and scale bars are in the units of substitutions per site. See taxon definitions and GenBank accession numbers in Additional file 1: Table S1. Additional file 3:
**Manually-curated foamy virus polymerase protein alignment.**
Additional file 4L:
**Manually-curated foamy virus polymerase coding region alignment.**
