# Supplementary material for: Time dependency of foamy virus evolutionary rate estimates
Source: BMC Evol Biol. 2015 Jun 26;15:119. doi: 10.1186/s12862-015-0408-z (PMC4480597; doi:10.1186/s12862-015-0408-z)
Supplement: Additional file 1: Table S1. — GenBank accession numbers of foamy virus (FV) nucleotide sequences. Table S2. Foamy virus (FV) divergence dates (t estimates), node-to-tip total per-lineage nucleotide substitutions (s estimates), and average evolutionary rates ($\overline{r}$r¯ estimates). Table S3. Model fitting – relationship between average evolutionary rate estimate and measurement timescale. Table S4. Summary of the results from leave-one-out cross validation analyses. Table S5. Summary of the results from the examination of the effect of the time-dependent rate phenomenon on evolutionary timescale inference. Table S6. Coefficients of rate variation. [file 12862_2015_408_MOESM1_ESM.docx]

# Additional File 1

**Table S1. GenBank accession numbers of** **foamy virus (FV) nucleotide sequences.**

| **FV**† | **Accession number** | **Host** |
| --- | --- | --- |
| PFV‡ | Y07725 | West chimpanzee (*Pan troglodytes schweinfurthii*) |
| SFVcpz | U04327 | East chimpanzee (*Pan troglodytes verus*) |
| SFVbnb | EU527700 & EU527595 | Bonobo (*Pan paniscus*) |
| SFVgor | HM245790 | Gorilla (*Gorilla gorilla sp.*) |
| SFVora | AJ544579 | Orangutan (*Pongo pygmaeus*) |
| SFVagm | M74895 | African green monkey (*Cercopithecus aethiops*) |
| SFVmac | NC_010819 | Macaque (*Macaca mulatta*) |
| SFVspm | EU010385 | Spider monkey (*Ateles sp.*) |
| SFVmar | GU356395 | Common marmoset (*Callithrix jacchus*) |
| SFVsqu | GU356394 | Squirrel monkey (*Saimiri sciureus*) |
| PSFVgal | KM233624 | Galago (*Otolemur crassicaudatus panganiensis*) |
| BFV | U94514 | Cow (*Bos taurus*) |
| EFV | AF201902 | Horse (*Equus ferus caballus)* |
| FFV | Y08851 | Domestic cat (*Felis catus*) |

†Acronyms used in FV names: PFV, prototype FV; SFVcpz, chimpanzee FV; SFVbnb, bonobo FV; SFVgor, gorilla FV; SFVora, orangutan FV; SFVagm, African green monkey FV; SFVmac, macaque FV; SFVmar, common marmoset FV; SFVspm, spider monkey FV; SFVsqu, squirrel monkey FV; PSFVgal, galago prosimian FV; BFV, bovine FV; EFV, equine FV; FFV, feline FV.

‡ Although PFV was isolated from a human [1] it is well-established that it in fact has a West chimpanzee origin [2-4]. It is therefore treated as a West chimpanzee FV here, which diverged from SFVcpz about 0.96 [95% HPD: 0.70-1.22] million years ago, inferred under the FV-host co-speciation assumption, based on the divergence date of *Pan troglodytes schweinfurthii* and *Pan troglodytes verus* [5].

1. Achong BG, Mansell PW, Epstein MA, Clifford P: **An unusual virus in cultures from a human nasopharyngeal carcinoma.** J Natl Cancer Inst 1971, **46**:299–307.

2. Switzer WM, Bhullar V, Shanmugam V, Cong M-E, Parekh B, Lerche NW, Yee JL, Ely JJ, Boneva R, Chapman LE, Folks TM, Heneine W: **Frequent simian foamy virus infection in persons occupationally exposed to nonhuman primates.** J Virol 2004, **78**:2780–9.

3. Meiering CD, Linial ML: **Historical perspective of foamy virus epidemiology and infection.** Clin Microbiol Rev 2001, **14**:165–76.

4. Liu W, Worobey M, Li Y, Keele BF, Bibollet-Ruche F, Guo Y, Goepfert PA, Santiago ML, Ndjango J-BN, Neel C, Clifford SL, Sanz C, Kamenya S, Wilson ML, Pusey AE, Gross-Camp N, Boesch C, Smith V, Zamma K, Huffman MA, Mitani JC, Watts DP, Peeters M, Shaw GM, Switzer WM, Sharp PM, Hahn BH: **Molecular ecology and natural history of simian foamy virus infection in wild-living chimpanzees.** PLoS Pathog 2008, **4**:e1000097.

5. Stone AC, Battistuzzi FU, Kubatko LS, Perry GH, Trudeau E, Lin H, Kumar S: **More reliable estimates of divergence times in Pan using complete mtDNA sequences and accounting for population structure.** Philos Trans R Soc Lond B Biol Sci 2010, **365**:3277–88.

**Table S2. Foamy virus (FV) divergence dates (**$\boldsymbol{t}$ **estimates), node-to-tip total per-lineage nucleotide substitutions (**$\boldsymbol{s}$ **estimates), and average evolutionary rates (**$\bar{\boldsymbol{r}}$ **estimates)**.

| **Node†** | $\boldsymbol{t}$ **(million years)** | | | **Median** $\boldsymbol{s}$ **(substitutions per site)**  **(95% HPD)** | **Median** $\bar{\boldsymbol{r}}$ **(substitutions per site per million years)**  **(95% HPD)** |
| --- | --- | --- | --- | --- | --- |
|  | **Median‡**  **(95% HPD)** | **Standard error** | **Reference**** |  |  |
| **1** | 0.96  (0.70,1.22) | 0.133 | [1] | 7.12E-02  (6.32E-02,7.91E-02) | 0.0748  (0.0546,0.0996) |
| **2** | 2.17  (1.28,3.21) | 0.531 | [2] | 0.117  (0.103,0.133) | 0.0539  (0.0305,0.0861) |
| **3** | 8.30  (6.58,10.07) | 0.903 |  | 0.167  (0.153,0.182) | 0.0201  (0.0159,0.0251) |
| **4** | 11.50  (9.18,13.58) | 1.199 |  | 0.145  (0.131,0.161) | 0.0127  (0.0097,0.0156) |
| **5** | 16.52  (13.45,19.68) | 1.610 |  | 0.231  (0.213,0.250) | 0.0141  (0.0113,0.017) |
| **6** | 31.56  (22.66,37.88) | 3.225 |  | 0.253  (0.234,0.271) | 0.0081  (0.0066,0.0099) |
| **7** | 43.47  (38.55,48.36) | 2.510 |  | 0.439  (0.405,0.476) | 0.0101  (0.0088,0.0116) |
| **8** | 87.18  (75.90,98.64) | 5.847 |  | 0.520  (0.477,0.565) | 0.006  (0.0052,0.0069) |
| **9** | 87.3  (85.3,88.8) | 1.020 | [3] | 0.390  (0.350,0.435) | 0.0045  (0.004,0.005) |
| **10** | 88.7  (86.7,90.7) | 1.020 |  | 0.514  (0.464,0.565) | 0.0058  (0.0052,0.0064) |
| **11** | 98.9  (96.2,101.6) | 1.378 |  | 0.558  (0.514,0.603) | 0.0056  (0.0052,0.0062) |
| **I*** | N/A | N/A | N/A | 0.246  (0.222,0.270) | N/A |
| **II*** | N/A | N/A | N/A | 0.336  (0.307,0.369) | N/A |

N/A= Not applicable

HPD= Highest probability density interval

† Referring to node numbers in blue on the FV tree in **fig. 1**.

‡ FV divergence dates were inferred directly from those of their hosts. The references refer to the host date references.

* We could not infer the $t$s for node **I**, and **II**, since they could not be mapped conclusively onto the host tree (**fig. 1**). As a result, both of them were excluded from the analysis of the relationship of average evolutionary rate estimate and the measurement timescale.

** 1. Stone AC, Battistuzzi FU, Kubatko LS, Perry GH, Trudeau E, Lin H, Kumar S: **More reliable estimates of divergence times in Pan using complete mtDNA sequences and accounting for population structure.** *Philos Trans R Soc Lond B Biol Sci* 2010, **365**:3277–88.

2. Perelman P, Johnson WE, Roos C, Seuánez HN, Horvath JE, Moreira MAM, Kessing B, Pontius J, Roelke M, Rumpler Y, Schneider MPC, Silva A, O’Brien SJ, Pecon-Slattery J: **A molecular phylogeny of living primates.** *PLoS Genet* 2011, **7**:e1001342.

3. Bininda-Emonds ORP, Cardillo M, Jones KE, MacPhee RDE, Beck RMD, Grenyer R, Price SA, Vos RA, Gittleman JL, Purvis A: **The delayed rise of present-day mammals.** *Nature* 2007, **446**:507–12.

**Table S3. Model fitting – relationship between average evolutionary rate estimate and measurement timescale.**

| **Model** | | | **Median** $\bar{R}^{2}$ **(95% HPD)** |
| --- | --- | --- | --- |
| **Model** | **Parameter** | **Median value (95% HPD)** |  |
| **Eq.9** | $\boldsymbol{\alpha}_{\boldsymbol{VEX}}$ | 0.1022 (0.0702,0.1460) | 0.99 (0.95,1.00) |
|  | $\boldsymbol{\beta}_{\boldsymbol{VEX}}$ | 0.7576 (0.4836,1.3329) |  |
|  | $\boldsymbol{k}_{\boldsymbol{VEX}}$ | 0.0043 (0.0030,0.0056) |  |
| **Eq.10** | $\boldsymbol{\alpha}_{\boldsymbol{EX}}$ | 0.0968 (0.0692,0.1351) | 0.97 (0.94,0.99) |
|  | $\boldsymbol{\beta}_{\boldsymbol{EX}}$ | 0.5357 (0.3549,0.9159) |  |
| **Eq.11** | $\boldsymbol{\alpha}_{\boldsymbol{VPL}}$ | 0.0299 (0.0223,0.0341) | 0.98 (0.92,1.00) |
|  | $\boldsymbol{\beta}_{\boldsymbol{VPL}}$ | 0.6044 (0.5497,0.6972) |  |
|  | $\boldsymbol{k}_{\boldsymbol{VPL}}$ | 0.0002 (0.0000,0.0021) |  |
| **Eq.12** | $\boldsymbol{\alpha}_{\boldsymbol{PL}}$ | 0.0304 (0.0274,0.0338) | 0.9819 (0.93,1.00) |
|  | $\boldsymbol{\beta}_{\boldsymbol{PL}}$ | 0.5982 (0.5490,0.6495) |  |

HPD= Highest probability density interval **Table S4. Summary of the results from leave-one-out cross validation analyses.**

| **Node** | **Model** | **Median reference** $\boldsymbol{t}$ **(95% HPD)** | **Median recovered** $\boldsymbol{t}$ **(95% HPD)** |
| --- | --- | --- | --- |
|  |  |  |  |
| **1** | **Eq.5** | 0.96 (0.70,1.22) | 2.08 (0.40,2.67) |
|  | **Eq.6** |  | 7.24 (6.32,8.31) |
|  | **Eq.7** |  | 0.02 (0.00,0.39) |
|  | **Eq.8** |  | 3.66 (2.59,4.86) |
| **2** | **Eq.5** | 2.17 (1.28,3.21) | 5.01 (1.98,6.59) |
|  | **Eq.6** |  | 12.53 (10.78,14.59) |
|  | **Eq.7** |  | 3.34 (1.21,5.92) |
|  | **Eq.8** |  | 8.48 (6.33,10.98) |
| **3** | **Eq.5** | 8.30 (6.58,10.07) | 11.49 (8.30,15.55) |
|  | **Eq.6** |  | 18.72 (16.47,20.92) |
|  | **Eq.7** |  | 12.18 (9.20,15.75) |
|  | **Eq.8** |  | 14.99 (12.34,17.93) |
| **4** | **Eq.5** | 11.50 (9.18,13.58) | 5.25 (2.32,8.50) |
|  | **Eq.6** |  | 15.68 (13.86,18.01) |
|  | **Eq.7** |  | 7.63 (4.71,10.75) |
|  | **Eq.8** |  | 11.34 (8.58,13.85) |
| **5** | **Eq.5** | 16.52 (13.45,19.68) | 26.87 (23.11,30.33) |
|  | **Eq.6** |  | 27.95 (25.12,30.81) |
|  | **Eq.7** |  | 26.23 (22.70,29.81) |
|  | **Eq.8** |  | 25.63 (22.38,29.09) |
| **6** | **Eq.5** | 31.56 (22.66,37.88) | 27.43 (22.36,32.20) |
|  | **Eq.6** |  | 29.36 (26.51,32.14) |
|  | **Eq.7** |  | 28.93 (24.71,32.34) |
|  | **Eq.8** |  | 26.73 (23.41,30.26) |
| **7** | **Eq.5** | 43.47 (38.55,48.36) | 74.50 (68.56,79.90) |
|  | **Eq.6** |  | 68.87 (63.00,74.22) |
|  | **Eq.7** |  | 73.90 (68.24,79.78) |
|  | **Eq.8** |  | 71.27 (65.71,77.50) |
| **8** | **Eq.5** | 87.18 (75.90,98.64) | 88.42 (82.22,95.63) |
|  | **Eq.6** |  | 86.75 (77.13,96.94) |
|  | **Eq.7** |  | 87.91 (81.62,94.97) |
|  | **Eq.8** |  | 88.74 (81.05,97.37) |
| **9** | **Eq.5** | 87.3 (85.3,88.8) | 43.06 (33.62,58.74) |
|  | **Eq.6** |  | 44.22 (37.18,51.98) |
|  | **Eq.7** |  | 48.85 (37.14,60.74) |
|  | **Eq.8** |  | 48.34 (39.99,56.48) |
| **10** | **Eq.5** | 88.7 (86.7,90.7) | 85.95 (77.65,95.63) |
|  | **Eq.6** |  | 83.61 (73.02,97.00) |
|  | **Eq.7** |  | 85.56 (77.17,94.87) |
|  | **Eq.8** |  | 85.83 (75.66,96.88) |
| **11** | **Eq.5** | 98.9 (96.2,101.6) | 95.53 (88.57,101.63) |
|  | **Eq.6** |  | 103.55 (91.18,120.98) |
|  | **Eq.7** |  | 95.30 (89.09,101.80) |
|  | **Eq.8** |  | 99.09 (90.71,107.13) |

| **Node** | **Model** | **Median MSE_OOS_ (95% HPD)** | **Mean MSE_OOS_ rank (95% HPD)** | **Mean rank different (95% confident interval)** | | | |
| --- | --- | --- | --- | --- | --- | --- | --- |
|  |  |  |  | **Eq.5** | **Eq.6** | **Eq.7** | **Eq.8** |
| **Overall** | **Eq.5** | 287.52 (169.43,400.58) | 3.32 | - | 0.14 (0.02,0.27) | 1.45 (1.33,1.58) | 1.70 (1.58,1.83) |
|  | **Eq.6** | 282.79 (204.40,369.84) | 3.18 |  | - | 1.31 (1.18,1.43) | 1.56 (1.43,1.68) |
|  | **Eq.7** | 239.04 (148.06,357.06) | 1.87 |  |  | - | 0.25 (0.13,0.38) |
|  | **Eq.8** | 236.25 (146.21,325.40) | 1.62 |  |  |  | - |

HPD= Highest probability density interval;

MSE_OOS_ = out-of-sample mean squared error**Table S5. Summary of the results from the examination of the effect of the time-dependent rate phenomenon on evolutionary timescale inference.**

| **Calibration scheme** | | **Node** | **Median evolutionary timescale (95% HPD)** | | | | |
| --- | --- | --- | --- | --- | --- | --- | --- |
|  |  |  | **Reference timescale** | **Molecular clock** | | | |
|  |  |  |  | **Power-law rate decay model** | **Strict clock** | **Log-normal relaxed clock** | **Random-local relaxed clock** |
| **Aggregated-node calibration scheme** | **Shallow-timescale calibration scheme** | **1** | 0.96  (0.70,1.22) | 0.49 (<0.01,1.06) | 1.21 (0.98,1.44) | 1.17 (0.94,1.41) | 1.20 (0.95,1.44) |
|  |  | **2** | 2.17  (1.28,3.21) | 2.48 (1.36,3.57) | 2.06 (1.61,2.54) | 2.38 (1.72,3.12) | 2.11 (1.55,3.07) |
|  |  | **3** | 8.30  (6.58,10.07) | 8.21 (6.54,9.84) | 3.01 (2.38,3.71) | 3.92 (2.48,6.67) | 3.09 (2.20,5.35) |
|  |  | **4** | 11.50  (9.18,13.58) | 5.06 (2.80,7.36) | 2.57 (1.99,3.18) | 3.22 (1.96,5.56) | 2.65 (1.80,4.95) |
|  |  | **5** | 16.52  (13.45,19.68) | 24.10 (11.88,59.72) | 4.11 (3.24,5.07) | 5.07 (3.30,8.43) | 4.21 (2.93,7.58) |
|  |  | **6** | 31.56  (22.66,37.88) | 32.30 (10.26,99.23) | 4.49 (3.51,5.49) | 5.65 (3.66,9.42) | 4.60 (3.28,8.50) |
|  |  | **8** | 87.18  (75.90,98.64) | 353.94 (32.70,6179.40) | 9.23 (7.24,11.41) | 11.36 (7.45,17.72) | 9.47 (6.69,18.05) |
|  |  | **10** | 88.7  (86.7,90.7) | 344.19 (29.77,6390.40) | 9.13 (7.15,11.32) | 11.01 (7.17,17.37) | 9.40 (6.50,17.90) |
|  |  | **11** | 98.9  (96.2,101.6) | 448.82 (34.44,9433.20) | 9.92 (7.78,12.18) | 12.15 (7.86,18.92) | 10.18 (7.25,19.51) |
|  | **Intermediate-timescale calibration scheme** | **1** | 0.96  (0.70,1.22) | 2.27 (<0.01,4.98) | 5.79 (4.91,6.83) | 5.76 (4.47,7.12) | 5.80 (4.86,6.80) |
|  |  | **2** | 2.17  (1.28,3.21) | 5.95 (1.54,10.06) | 9.50 (7.93,11.28) | 9.69 (7.85,11.81) | 9.51 (7.92,11.30) |
|  |  | **3** | 8.30  (6.58,10.07) | 12.00 (6.53,16.06) | 13.57 (11.69,15.54) | 13.87 (11.63,16.13) | 13.60 (11.80,15.66) |
|  |  | **4** | 11.50  (9.18,13.58) | 9.05 (4.69,12.55) | 11.82 (10.23,13.36) | 11.95 (10.21,13.70) | 11.85 (10.28,13.46) |
|  |  | **5** | 16.52  (13.45,19.68) | 22.50 (19.21,26.42) | 18.72 (16.40,21.05) | 18.50 (16.08,20.95) | 18.67 (16.36,21.03) |
|  |  | **6** | 31.56  (22.66,37.88) | 27.24 (21.36,33.22) | 20.73 (18.18,23.45) | 21.02 (18.17,23.92) | 20.71 (18.09,23.34) |
|  |  | **8** | 87.18  (75.90,98.64) | 112.20 (45.77,379.30) | 42.42 (36.33,48.31) | 43.10 (35.89,50.51) | 42.44 (36.53,48.55) |
|  |  | **10** | 88.7  (86.7,90.7) | 112.71 (44.75,385.60) | 41.86 (35.63,48.32) | 42.05 (34.08,50.76) | 42.04 (35.62,48.80) |
|  |  | **11** | 98.9  (96.2,101.6) | 129.78 (50.05,525.41) | 45.49 (39.27,51.72) | 46.04 (38.39,54.25) | 45.63 (39.16,52.09) |
|  | **Deep-timescale calibration scheme** | **1** | 0.96  (0.70,1.22) | 4.83 (<0.01,11.49) | 12.53 (11.10,14.09) | 12.29 (9.90,14.79) | 12.49 (10.99,14.05) |
|  |  | **2** | 2.17  (1.28,3.21) | 9.8748 (0.6801,19.88) | 20.5608 (17.9308,23.4802) | 20.6399 (17.068,24.3968) | 20.5134 (17.7156,23.2569) |
|  |  | **3** | 8.30  (6.58,10.07) | 16.532 (2.9957,29.333) | 29.3776 (26.8051,32.0309) | 29.6195 (25.581,33.6921) | 29.2913 (26.7465,32.0123) |
|  |  | **4** | 11.50  (9.18,13.58) | 13.691 (1.307,24.298) | 25.538 (23.1007,28.2975) | 25.7833 (21.1277,30.4173) | 25.4939 (22.9794,28.4543) |
|  |  | **5** | 16.52  (13.45,19.68) | 27.065 (8.7927,41.573) | 40.6602 (37.5502,43.7963) | 39.7908 (35.3096,44.798) | 40.54 (37.2333,43.9428) |
|  |  | **6** | 31.56  (22.66,37.88) | 30.81 (11.92,45.538) | 44.4404 (41.2962,47.6274) | 44.3478 (39.809,49.5427) | 44.3584 (41.0711,47.6097) |
|  |  | **8** | 87.18  (75.90,98.64) | 87.848 (78.936,94.61) | 91.6311 (87.1192,96.6365) | 92.1123 (86.6513,97.7652) | 91.5347 (86.8092,96.5106) |
|  |  | **10** | 88.7  (86.7,90.7) | 87.537 (82.249,93.424) | 88.9656 (87.0212,90.7901) | 88.8538 (86.9493,90.8281) | 88.9908 (87.0456,90.7739) |
|  |  | **11** | 98.9  (96.2,101.6) | 98.332 (94.915,101.75) | 98.518 (96.7113,100.3155) | 98.5842 (96.7405,100.4127) | 98.5135 (96.7048,100.3027) |

HPD= Highest probability density interval

| **Calibration scheme** | | **Node** | **Median evolutionary timescale (95% HPD)** | | | | |
| --- | --- | --- | --- | --- | --- | --- | --- |
|  |  |  | **Reference timescale** | **Molecular clock** | | | |
|  |  |  |  | **Power-law rate decay model** | **Strict clock** | **Log-normal relaxed clock** | **Random-local relaxed clock** |
| **Dispersed-node calibration scheme** | **Dispersed-I calibration scheme** | **1** | 0.96  (0.70,1.22) | 1.36 (0.76,2.12) | 3.95 (3.27,4.63) | 1.71 (0.77,2.66) | 1.71 (1.13,2.28) |
|  |  | **2** | 2.17  (1.28,3.21) | 3.82 (2.39,5.38) | 5.43 (4.66,6.17) | 2.97 (2.04,3.92) | 2.76 (1.91,3.62) |
|  |  | **3** | 8.30  (6.58,10.07) | 8.01 (5.94,10.19) | 9.78 (8.09,11.51) | 10.10 (4.78,15.50) | 4.14 (2.81,5.57) |
|  |  | **4** | 11.50  (9.18,13.58) | 5.97 (4.23,8.13) | 8.74 (7.10,10.40) | 12.56 (4.13,23.38) | 21.00 (16.94,25.63) |
|  |  | **5** | 16.52  (13.45,19.68) | 15.78 (12.96,18.19) | 14.00 (11.61,16.32) | 16.67 (13.57,19.68) | 18.20 (14.74,21.33) |
|  |  | **6** | 31.56  (22.66,37.88) | 18.94 (15.77,21.99) | 15.26 (12.67,17.94) | 22.26 (15.29,35.73) | 34.31 (27.78,42.18) |
|  |  | **8** | 87.18  (75.90,98.64) | 85.76 (74.43,94.55) | 33.05 (26.88,39.02) | 81.12 (68.96,92.92) | 77.62 (65.64,90.30) |
|  |  | **10** | 88.7  (86.7,90.7) | 85.12 (72.03,100.21) | 31.38 (25.79,37.54) | 69.31 (35.54,95.99) | 76.89 (63.09,91.99) |
|  |  | **11** | 98.9  (96.2,101.6) | 100.18 (85.63,113.60) | 34.58 (28.48,40.88) | 84.77 (69.67,101.08) | 83.20 (69.54,97.74) |
|  | **Dispersed-II calibration scheme** | **1** | 0.96  (0.70,1.22) | 3.23 (2.22,4.44) | 2.60 (2.41,2.78) | 1.06 (0.81,1.31) | 1.01 (0.74,1.26) |
|  |  | **2** | 2.17  (1.28,3.21) | 7.32 (5.33,9.69) | 9.97 (8.22,11.73) | 7.76 (2.20,17.10) | 7.02 (4.93,10.05) |
|  |  | **3** | 8.30  (6.58,10.07) | 13.35 (10.66,16.43) | 16.73 (15.05,18.62) | 14.77 (5.70,28.03) | 11.89 (8.98,15.30) |
|  |  | **4** | 11.50  (9.18,13.58) | 10.50 (8.74,12.46) | 14.97 (13.64,16.34) | 11.53 (9.17,13.75) | 11.58 (9.24,13.99) |
|  |  | **5** | 16.52  (13.45,19.68) | 22.90 (19.14,26.64) | 25.69 (23.39,27.98) | 22.64 (10.41,39.46) | 17.39 (12.97,21.85) |
|  |  | **6** | 31.56  (22.66,37.88) | 26.66 (22.69,30.07) | 28.10 (25.83,30.37) | 28.40 (14.39,48.93) | 19.58 (14.99,24.13) |
|  |  | **8** | 87.18  (75.90,98.64) | 89.30 (79.06,98.89) | 69.45 (64.53,74.50) | 78.49 (46.80,101.56) | 42.95 (33.03,53.58) |
|  |  | **10** | 88.7  (86.7,90.7) | 88.63 (86.65,90.40) | 85.27 (83.24,87.26) | 88.56 (86.55,90.56) | 88.59 (86.55,90.57) |
|  |  | **11** | 98.9  (96.2,101.6) | 100.88 (92.34,109.61) | 85.64 (83.40,87.69) | 92.20 (86.60,107.48) | 96.78 (87.79,105.25) |
|  | **Dispersed-III calibration scheme** | **1** | 0.96  (0.70,1.22) | 3.48 (2.06,4.92) | 8.73 (7.80,9.75) | 4.16 (1.99,6.34) | 3.77 (3.01,4.63) |
|  |  | **2** | 2.17  (1.28,3.21) | 7.72 (5.31,10.60) | 13.87 (12.33,15.52) | 7.06 (4.88,9.11) | 6.18 (4.84,7.56) |
|  |  | **3** | 8.30  (6.58,10.07) | 13.89 (10.85,16.93) | 17.51 (16.36,18.63) | 9.46 (7.65,11.18) | 8.76 (7.16,10.38) |
|  |  | **4** | 11.50  (9.18,13.58) | 10.99 (7.70,14.07) | 20.56 (18.45,22.68) | 16.26 (6.07,27.07) | 24.04 (21.19,26.89) |
|  |  | **5** | 16.52  (13.45,19.68) | 23.55 (19.57,27.69) | 31.33 (28.91,33.91) | 23.69 (14.04,32.15) | 11.79 (9.40,14.20) |
|  |  | **6** | 31.56  (22.66,37.88) | 27.20 (23.23,31.10) | 34.95 (32.48,37.37) | 30.77 (25.04,37.05) | 38.77 (34.93,42.38) |
|  |  | **8** | 87.18  (75.90,98.64) | 88.36 (80.87,95.03) | 83.44 (77.77,89.09) | 88.59 (64.16,100.07) | 89.70 (84.14,95.36) |
|  |  | **10** | 88.7  (86.7,90.7) | 87.83 (81.24,95.62) | 88.71 (82.41,95.28) | 89.89 (57.64,100.38) | 92.06 (86.24,98.15) |
|  |  | **11** | 98.9  (96.2,101.6) | 99.58 (97.21,102.14) | 96.82 (94.86,98.66) | 98.74 (96.76,100.61) | 98.55 (96.73,100.50) |

HPD= Highest probability density interval

**Table S6. Coefficients of rate variation.**

| **Calibration scheme** | | **Mean coefficients of rate variation (95%HPD)** | |
| --- | --- | --- | --- |
|  |  | **Log-normal relaxed clock** | **Random-local relaxed clock** |
| **Aggregated-node** | **Shallow-timescale** | 0.197 (0.065-0.430) | 0.017 (<0.001-0.091) |
|  | **Intermediate-timescale** | 0.112 (0.048-0.183) | 0.011 (<0.001-0.062) |
|  | **Deep-timescale** | 0.106 (0.041-0.172) | 0.009 (<0.001-0.057) |
| **Dispersed-node** | **Dispersed-I** | 0.940 (0.648-1.288) | 1.004 (0.795-1.182) |
|  | **Dispersed-II** | 1.216 (0.922-1.517) | 1.122 (0.935-1.321) |
|  | **Dispersed-III** | 0.665 (0.482-0.911) | 0.402 (0.360-0.452) |

HPD= Highest probability density interval
