# Supplementary figures and images for: Time dependency of foamy virus evolutionary rate estimates
Source: BMC Evol Biol. 2015 Jun 26;15:119. doi: 10.1186/s12862-015-0408-z (PMC4480597; doi:10.1186/s12862-015-0408-z)

**Pol protein****Bayesian phylogenies**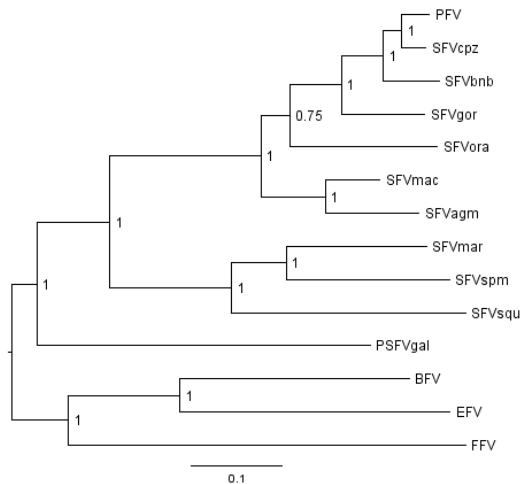**Pol nucleotide**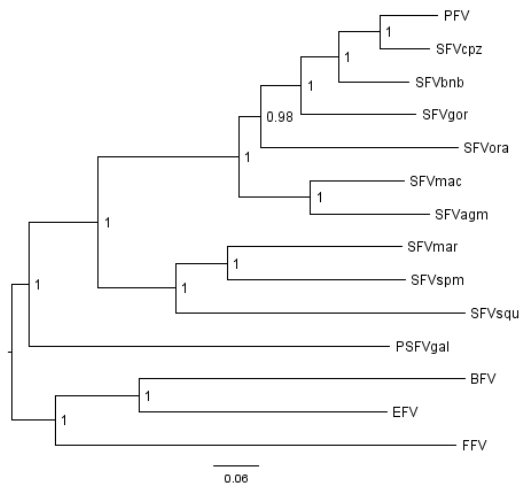**Maximum likelihood phylogenies**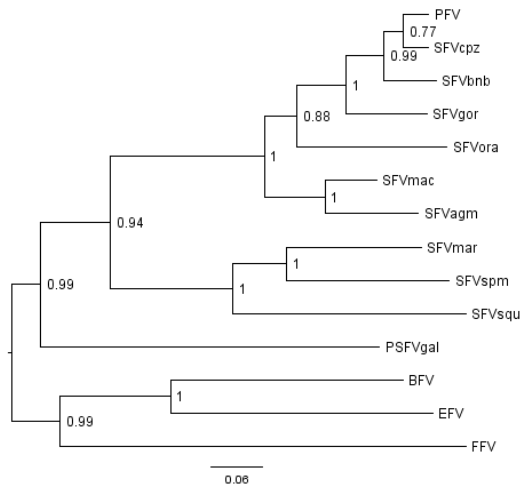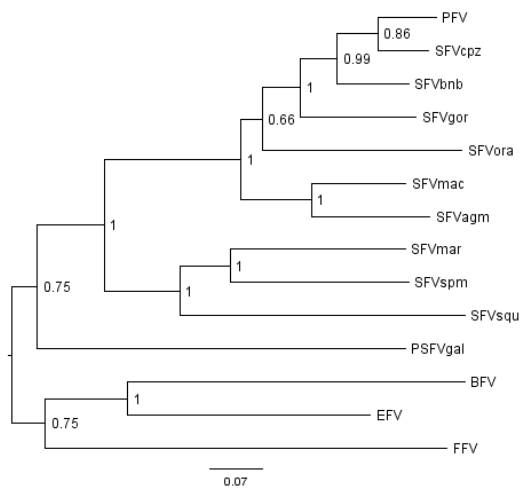

Supplement: Additional file 2: Figure S1. — Foamy virus (FV) phylogenies. Four phylogenies of 14 extant FVs were constructed based on Pol protein (left column) and pol nucleotide (right column) alignments under the Bayesian (top row) and maximum-likelihood (bottom row) framework (See Methods for details). Numbers on nodes are node supports (top row: Bayesian posterior support; bottom row: bootstrap support), and scale bars are in the units of substitutions per site. See taxon definitions and GenBank accession numbers in Additional file 1: Table S1. [file 12862_2015_408_MOESM2_ESM.pdf]
